# Diagnostic Potential of Urine CXCL10 and Donor-Derived cfDNA in Kidney Transplant Rejection

**DOI:** 10.3389/ti.2026.15517

**Published:** 2026-03-27

**Authors:** Daniel Fantus, Robert Balshaw, Chee Loong Saw, Majda Belkaid, Narin S. Tangprasertchai, Thierry Viard, François Gougeon, Justin Belair, Claude Daniel, Caroline Lamarche, Sílvia Casas, Heloise Cardinal, Julie Ho

**Affiliations:** 1 Division of Nephrology, Department of Medicine, Centre Hospitalier de l’Université de Montréal (CHUM), Montreal, QC, Canada; 2 Centre de Recherche de CHUM (CRCHUM), Montreal, QC, Canada; 3 George and Fay Yee Centre for Healthcare Innovation, Rady Faculty of Health Sciences, University of Manitoba, Winnipeg, MB, Canada; 4 Histocompatiblility and Immunogenetics Laboratory, McGill University Health Center (MUHC), Montreal, QC, Canada; 5 CareDx Inc., Brisbane, CA, United States; 6 Department of Pathology and Cell Biology, Centre Hospitalier de l’Université de Montréal (CHUM), Montreal, QC, Canada; 7 Division of Nephrology, Centre de Recherche de l’Hopital Maisonneuve-Rosemont, Montreal, QC, Canada; 8 Department of Internal Medicine, Rady Faculty of Health Sciences, University of Manitoba, Winnipeg, MB, Canada

**Keywords:** CXCL10, dd-cfDNA, kidney transplantation, non-invasive immune monitoring, rejection

## Abstract

Data suggests donor-derived cell-free DNA (dd-cfDNA) and urine CXCL10 outperform serum creatinine as a biomarker of antibody-mediated rejection (AMR) and T cell-mediated rejection (TCMR). We hypothesized that combining these biomarkers would improve the overall detection of rejection. We performed a retrospective two-center, case-controlled study of 103 adult renal transplant recipients who had for-cause or surveillance biopsies with corresponding urine and plasma samples. Rejection was classified by Banff 2022 criteria. While log_10_%dd-cfDNA correlated more strongly than log_10_CXCL10 with glomerulitis (r = 0.55, p < 0.001 vs. r = 0.25, p = 0.01) and peritubular capillaritis (r = 0.47, p < 0.001 vs. r = 0.23, p = 0.02), log_10_CXCL10 was a better correlate of tubulitis (r = 0.28, p = 0.004 vs. r = 0.054, p = 0.59). Both dd-cfDNA > 0.5% (OR 21.9, 95% CI 3.74–180, p < 0.001) and *de novo* DSA (OR 10.4, 95% CI 1.16–157, p = 0.037) were independently associated with AMR vs. no rejection (NR), while log_10_ serum creatinine and log_10_CXCL10 were not (p > 0.05). While dd-cfDNA >0.5% (OR 5.37, 95% CI 1.04–31.5, p = 0.047) was independently associated with Banff ≥1A TCMR vs. NR, log_10_CXCL10 was a significant predictor of TCMR in a model without %dd-cfDNA (OR 3.12, 95% CI 1.09–10.4, p = 0.043). Biomarker-guided screening strategies based on dd-cfDNA and urine chemokines such as CXCL10 for AMR (microvascular injury) and TCMR (tubulitis) warrant further study.

## Graphical Abstract

**Figure:**
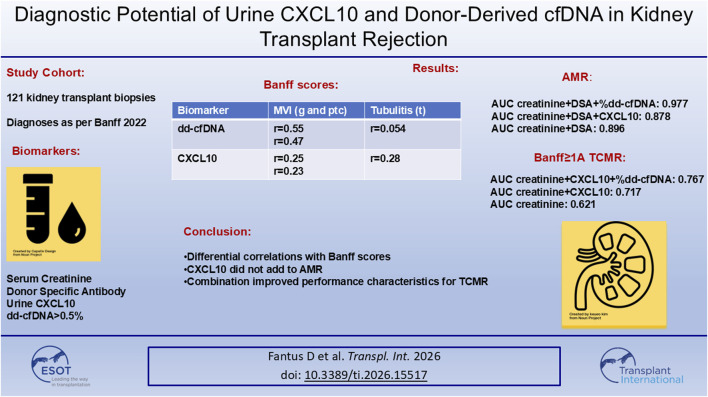

## Introduction

Despite the widespread use of modern immunosuppressive drugs, alloimmune injury remains the most common cause of renal allograft failure today [1–6]. While a kidney biopsy remains the gold standard for rejection diagnosis, they are invasive, resource-intensive, subject to sampling error, and unsuited for longitudinal monitoring. Despite the need for non-invasive biomarkers, serum creatinine and donor specific antibody (DSA) remain the primary non-invasive tests of alloimmune renal injury used clinically [7]. Serum creatinine is a poor diagnostic biomarker of rejection [8, 9] and often only increases after significant allograft damage has occurred. DSA can be absent despite microvascular injury [10, 11], and detection of *de novo* DSA is only 50% predictive of subclinical AMR [12]. A novel non-invasive biomarker should be able to rule out rejection and accurately detect rejection early [13].

Urine C-X-C motif chemokine ligand 10 (CXCL10) is an interferon-gamma induced, CXC chemokine receptor 3 (CXCR3) chemokine that is highly expressed by infiltrating leukocytes and renal tubules and plays an important role in leukocyte trafficking and T helper cell type 1 (Th1) responses. Studies have shown that allograft inflammation from a variety of causes including BK viremia and urinary tract infection can upregulate urine CXCL10 [14–16]. During acute rejection, urine CXCL10 rises prior to serum creatinine [8, 17], can detect subclinical rejection [18–20], decreases after treatment of rejection [21], and remains elevated in patients who develop allograft dysfunction [17, 21–24]. Furthermore, in an unselected kidney transplant population, CXCL10 outperformed serum creatinine-based monitoring [20, 25]. However, urine CXCL10 largely reflects inflammation in the tubulointerstitial compartment [24]. As a result, arteritis and/or glomerulitis may be missed by this monitoring strategy.

Donor-derived cell-free DNA (dd-cfDNA) is a non-invasive biomarker of allograft injury that can be measured by assaying a preselected number of bi-allelic single nucleotide polymorphisms (SNPs) to distinguish donor vs. recipient cell-free DNA (cfDNA) in a sample extracted from plasma [26]. Cell death due to apoptosis and necrosis is thought to be the principal driver of cfDNA, including dd-cfDNA, release [27]. Multiple studies have shown that dd-cfDNA strongly correlates with rejection and even more strongly with AMR [28–32]. Further, dd-cfDNA has been show to increase between 1 and 5 months *before* biopsy-proven AMR [33] and decrease *after* treatment of rejection [34]. However, sensitivity is lower for TCMR, particularly low-grade and borderline TCMR [28, 35, 36].

Due to these different properties, we hypothesized that plasma dd-cfDNA and urine CXCL10 are complementary biomarkers. While elevated plasma dd-cfDNA reflects injury in the vascular compartment, urine CXCL10 reflects tubulointerstitial injury and inflammation. By directly comparing both the individual and combined diagnostic performance of plasma dd-cfDNA and urine CXCL10 when added to standard-of-care biomarkers (serum creatinine and DSA), we sought to assess how each biomarker complements rejection phenotypes.

## Materials and Methods

### Study Population

We performed a two-site, case-controlled study selecting 121 biopsies with corresponding pre-biopsy urine and plasma samples in our transplant biobank. This study was approved by the research ethics board of the Centre hospitalier de l’Université de Montréal on 29 August 2022 (REB number MP-02-2023-10920).

Inclusion criteria were adults (18 or more years of age) who received a renal allograft between February 2011 and June 2021 and underwent a for-cause or protocol biopsy. We excluded recipients who were pregnant, simultaneous dual-organ transplants, or patients who were prior solid non-renal organ or bone marrow transplant recipients. We also excluded patients with high-grade BK viremia ( >1000 copies/mL) and clinical cystitis/pyelonephritis at the time of biopsy, as both are associated with high urine CXCL10 levels. Our centers routinely perform one surveillance biopsy 3–6 months post-transplant.

The usual immunosuppressive protocol at our centers includes induction immunosuppression with either anti-thymocyte globulin or basiliximab and maintenance immunosuppression with mycophenolic acid, tacrolimus, and prednisone (see Table 1).

**TABLE 1:** Demographic characteristics of the overall selected cohort and by diagnostic subgroup (no rejection, borderline TCMR, Banff ≥1A TCMR, and AMR).

Characteristic	Overall N = 103^a^	No Rejection N = 53^a^	Borderline N = 18^a^	TCMR N = 15^a^	AMR N = 17^a^	p-value^b^
Recipient age at biopsy	52 [40, 61]	54 [41, 60]	54 [42,63]	53 [40,69]	42 [34,53]	0.093
Recipient male sex	72 (70%)	39 (74%)	12 (67%)	9 (60%)	12 (71%)	0.8
Cause of ESRD	​	​	​	​	​	0.2
Polycystic kidney disease	26 (29%)	13 (31%)	5 (31%)	3 (20%)	5 (31%)	​
Glomerulonephritis	25 (28%)	11 (26%)	2 (13%)	7 (47%)	5 (31%)	​
Diabetes	18 (20%)	12 (29%)	2 (13%)	3 (20%)	1 (6.3%)	​
Vascular	8 (9.0%)	2 (4.8%)	2 (13%)	1 (6.7%)	3 (19%)	​
Genetic	5 (5.6%)	1 (2.4%)	2 (13%)	0 (0%)	2 (13%)	​
Other	7 (7.9%)	3 (7.1%)	3 (19%)	1 (6.7%)	0 (0%)	​
Unknown	14 (13.6%)	11 (20.8%)	2 (11.1%)	0 (0%)	1 (5.9%)	​
Dialysis duration (months)	31 [13,59]	30 [13,54]	24 [15,65]	37 [15,72]	36 [12,59]	>0.9
Repeat transplant	5 (4.9%)	1 (1.9%)	0 (0%)	1 (6.7%)	3 (17.6%)	0.048
Donor age	51 [36, 57]	51 [40, 55]	54 [49, 64]	52 [39, 63]	35 [31, 52]	0.051
Donor male sex	53 (51%)	27 (51%)	11 (61%)	7 (47%)	8 (47%)	0.8
Living donor	25 (24%)	16 (30%)	2 (11%)	1 (6.7%)	6 (35%)	0.093
Delayed graft function	13 (13%)	4 (7.5%)	4 (22%)	5 (33%)	0 (0%)	0.009
Induction therapy	​	​	​	​	​	0.8
Basiliximab	79 (77%)	40 (75%)	13 (72%)	13 (87%)	13 (81%)	​
Anti-thymocyte globulin (ATG)	13 (13%)	8 (15%)	2 (11%)	2 (13%)	1 (6.3%)	​
Other	10 (9.8%)	5 (9.4%)	3 (17%)	0 (0%)	2 (13%)	​
Maintenance immunosuppression	​	​	​	​	​	0.023
Triple therapy (tacrolimus, mycophenolate and prednisone)	84 (82%)	47 (89%)	14 (78%)	11 (73%)	12 (71%)	​
Bitherapy (tacrolimus and either mycophenolate or prednisone)	9 (8.7%)	4 (7.5%)	2 (11%)	3 (20%)	1 (5.9%)	​
Other	10 (10%)	2 (3.8%)	2 (11%)	1 (6.7%)	4 (24%)	​
Surveillance biopsy	55 (53%)	44 (83%)	8 (44%)	2 (13%)	1 (5.9%)	<0.001
Months post-transplant	6 [4, 12]	6 [4, 12]	7 [4, 9]	3 [3, 9]	23 [8, 46]	0.001
Mean proteinuria (grams/L) (SD)	0.24 (0.61)	0.10 (0.25)	0.25 (0.44)	0.80 (1.65)	0.35 (0.44)	0.081
eGFR at biopsy (ml/min/1.73m^2^)	49 [38, 60]	52 [44, 62]	43 [28, 53]	47 [33, 54]	42 [36, 53]	0.050

^a^ Median [Q1, Q3]; n (%), SD, standard deviation.

^b^ Kruskal-Wallis rank sum test; Fisher’s Exact Test for Count Data with simulated p-value (based on 2000 replicates); Pearson’s Chi-squared test.

ESRD, end-stage renal disease. eGFR, estimated glomerular filtration rate.

Exclusion criteria included simultaneous multi-organ transplantation, prior non-renal organ or bone marrow transplantation, pregnancy, and incomplete biomarker data (urine CXCL10, plasma dd-cfDNA, and DSA). For those patients who underwent multiple biopsies, only the last biopsy was included. ESRD = end-stage renal disease, eGFR = estimated glomerular filtration rate in mL/min/1.73m^2^.

### Dependent Variables

Cases were defined as patients with diagnoses of AMR, Banff ≥1A TCMR, or borderline TCMR as classified by the Banff 2022 criteria [11]. Ambiguous classifications were adjudicated by a second nephrologist (JH) and pathologist (IG). Inadequate biopsies not graded by adhering to the Banff criteria were excluded. Biopsy-confirmed BK nephropathy, pyelonephritis, and recurrent glomerular disease were also excluded.

Per Banff 2022 criteria, the AMR category included all diagnoses of active AMR, probable AMR, microvascular inflammation (MVI), mixed rejection, and probable mixed rejection [11]. Diagnoses of chronic AMR were not excluded provided there was a concomitant active component as characterized by glomerulitis, peritubular capillaritis or C4d positivity. Borderline TCMR was defined using the i1t1 threshold. The biopsy-proven acute rejection (BPAR) category was a combination of the AMR and Banff ≥1A TCMR categories with borderline TCMR excluded.

Controls (NR) were defined as patients with either normal histology or low-grade inflammation that did not meet the current Banff diagnostic criteria for BPAR or borderline TCMR.

### Independent Variables

#### Urinary CXCL10

Urine samples were collected immediately prior to biopsy and supernatants processed and stored at −80 °C. Urine CXCL10 was measured at the Transplant Immunology Lab, Canadian Blood Services, University of Manitoba using the Meso Scale V-Plex assay, using previously reported methods [37]. The subclinical rejection threshold used to calculate sensitivity and specificity for urine CXCL10 was > 13 pg/mL for male recipients over 2 weeks post-transplant, > 33 pg/mL for female recipients from 2 weeks to 5 months post-transplant, and > 13 pg/mL for female recipients > 6 months post-transplant [37, 38]. Units of measurement were picograms per ml.

#### Donor Derived Cell-free DNA

cfDNA was extracted from EDTA plasma samples collected immediately prior to the biopsy, following a protocol designed to avoid genomic DNA contamination. The percent dd-cfDNA was determined using the AlloSeq cfDNA Assay and Software (CareDx, Brisbane, CA) by the manufacturer. Due to the significant number of borderline TCMR cases (n = 18) in our study, coupled with the fact that our centers perform protocol biopsies as part of standard care, we selected an injury threshold of 0.5%. Others have previously compared sensitivity and specificity at various thresholds and have found sensitivity for rejection higher at this threshold [36, 39, 40]. Prior comparison of this assay versus the Allosure kidney assay has shown high correlation [41].

#### Donor-Specific Antibodies

DSA was assessed in samples collected either at the time of biopsy or in the preceding month. Analysis was conducted at the HLA laboratory of the McGill University Health Centre using a threshold of 1000 mean fluorescence intensity (MFI) units of the immunodominant DSA to dichotomize DSA as positive vs. negative per Canadian standards [42].

### Statistical Analyses

Individual characteristics of the biopsies in each group were summarized within groups and overall using quartiles (Q1, median, and Q3) for numerical variables and counts and percentages for categorical variables. Kruskal-Wallis was used to compare the distribution of numeric variables between groups (or Wilcoxon tests for two-group comparisons). Chi-squared tests were used to compare categorical variables; Fisher’s Exact test was used when cell sizes were smaller than 5.

Logistic regression models were used to examine relationships between plasma %dd-cfDNA, urine CXCL10, serum creatinine, and rejection events both with and without consideration of DSA depending on the rejection phenotype. Generalized estimating equations were used in sensitivity analyses to fit similar models to a dataset incorporating all biopsies with complete data, assuming an exchangeable working correlation matrix to account for within-participant correlations. Due to skewness, plasma % dd-cfDNA, urine CXCL10, and serum creatinine concentrations were analyzed on a log_10_ scale. Their association with Banff scores were described using Pearson correlations. Sensitivity and specificity for %dd-cfDNA and urine CXCL10 using thresholds as described above are also reported. As the small number of AMR events led to difficulties fitting conventional maximum likelihood logistic regression models, parameter estimates for the AMR vs. NR model were obtained via Firth’s penalized likelihood method [43]. Differences in AIC (Akaike Information Criterion) values were used as a comparative measure of model fit, with lower AIC values indicating a better fitting model. Model discrimination was assessed by evaluating the AUC (area under the receiver-operator characteristic curve). Statistical analyses were performed using R (version 4.4.2) and the logistf package (version 1.26.0). In general, p-values less than 0.05 were described as statistically significant, without correction for multiple inference.

## Results

**FIGURE 1:**
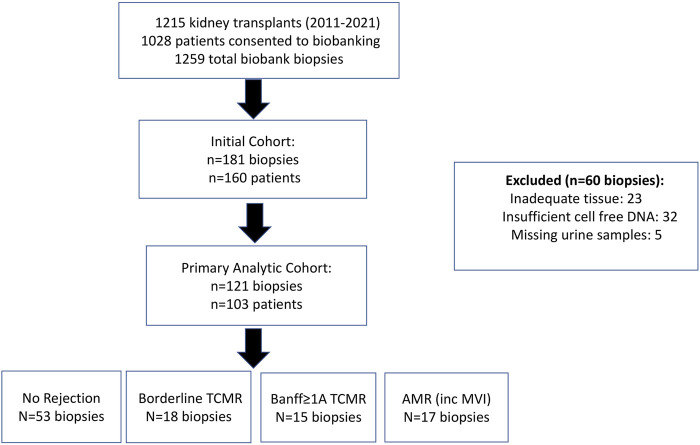
Study Population. Out of a total of 1259 biopsies performed during the study period as part of our biobank, 181 biopsies were initially selected from 160 patients. However, 60 biopsies were subsequently excluded due to incomplete biomarker data or an inadequate biopsy not adhering to the Banff criteria. The remaining 121 biopsies were selected from 103 patients with one of the following diagnoses as per Banff 2022 criteria: no rejection, borderline TCMR, Banff ≥1A TCMR, and AMR (including probable AMR and MVI). The primary analytic cohort encompassed 103 biopsies from 103 patients. inc = included.

Between 26 February 2011 and 8 June 2021, 1,215 patients underwent renal transplantation at our institutions (Figure 1). Of these patients, 1,028 enrolled in our biobank and 1,259 biopsies were performed. Although our initial cohort of cases and controls encompassed 181 biopsies from 160 patients, 60 biopsies were ultimately excluded due to inadequate tissue and/or incomplete Banff scores (n = 23), insufficient cell free DNA for analysis (n = 32), or urine samples that were not collected (n = 5). As a result, 121 biopsies from 103 patients had measurements for all traditional and novel biomarkers as well as sufficient tissue to provide Banff scores. Demographic characteristics, other than shorter dialysis exposure and cause of end-stage renal disease, were similar between our cohort and excluded patients (see Supplementary Table S1). As there was no significant difference in our results when we used a generalized estimating equation (GEE) model to account for multiple biopsies per patient, our primary analytic cohort comprised 103 biopsies: one per patient, with the most recent biopsy selected (see Supplementary Table S2; Supplementary Figure S1). Within this cohort, 17.5% (n = 18) of the biopsies were categorized as Banff Borderline TCMR, 14.6% (n = 15) were Banff ≥1A TCMR, and 16.5% (n = 17) were AMR/mixed rejection (see Table 1).

Demographic characteristics of the overall cohort and by diagnostic category are presented in Table 1; Supplementary Table S3. All patients underwent ABO-compatible transplantation and had a negative flow cytometric or complement-dependent cytotoxicity crossmatch prior to transplantation as well as no pre-transplant DSA. AMR patients tended to be younger and were less likely to have diabetes and delayed graft function. They were also less likely to have been treated with maintenance immunosuppression consisting of tacrolimus, MMF, and prednisone at the time of rejection. Although most of the TCMR and AMR biopsies were performed due to graft dysfunction, the AMR biopsies were generally performed later post-transplant.

**FIGURE 2:**
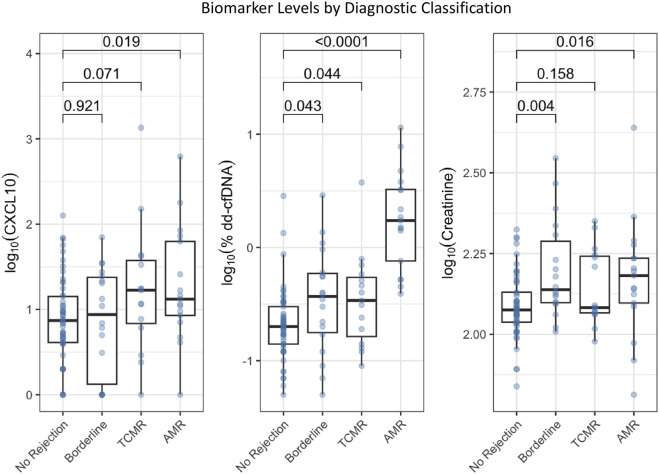
A comparison of log-transformed biomarker levels (urine CXCL10, plasma %dd-cfDNA, and serum creatinine) by diagnostic classification (no rejection, borderline, TCMR, and AMR).

Biomarker levels are presented in Table 2; Figure 2, stratified by diagnostic category. The distribution of Banff scores by rejection category is also provided in Table 2. Log_10_%dd-cfDNA is higher in patients with AMR while log_10_ serum creatinine and log_10_CXCL10 show smaller between-group differences (Figure 2).

**TABLE 2:** A comparison of biomarker levels and Banff scores by diagnostic classification.

Characteristic	No Rejection N = 53^a^	Borderline N = 18^a^	TCMR N = 15^a^	AMR N = 17^a^	Overall N = 103^a^	p-value^b^
Urine CXCL10 (pg/mL)	7 [4, 14]	9 [1, 25]	17 [6, 42]	13 [9, 63]	9 [4, 22]	0.070
CXCL10 positive	13 (25%)	8 (44%)	8 (53%)	9 (53%)	38 (37%)	0.056
Serum creatinine (μmol/L)	119 [109,135]	138 [125, 203]	121 [116, 176]	152 [125, 172]	125 [114, 159]	0.008
%dd-cfDNA	0.20 [0.14, 0.30]	0.37 [0.17, 0.60]	0.34 [0.14, 0.58]	1.73 [0.76, 3.25]	0.26 [0.17, 0.56]	<0.001
%dd-cfDNA positive^c^	3 (5.7%)	7 (39%)	5 (33%)	15 (88%)	30 (29%)	<0.001
DSA positive (*de novo*)	1 (1.9%)	0 (0%)	0 (0%)	12 (71%)	13 (13%)	<0.001
Glomerulitis (g score)	​	​	​	​	​	<0.001
0	53 (100%)	18 (100%)	15 (100%)	6 (35%)	92 (89%)	​
1	0 (0%)	0 (0%)	0 (0%)	6 (35%)	6 (5.8%)	​
2	0 (0%)	0 (0%)	0 (0%)	4 (24%)	4 (3.9%)	​
3	0 (0%)	0 (0%)	0 (0%)	1 (5.9%)	1 (1.0%)	​
Interstitial inflammation (i score)	​	​	​	​	​	<0.001
0	52 (98%)	0 (0%)	1 (6.7%)	10 (59%)	63 (61%)	​
1	1 (1.9%)	18 (100%)	3 (20%)	2 (12%)	24 (23%)	​
2	0 (0%)	0 (0%)	9 (60%)	3 (18%)	12 (12%)	​
3	0 (0%)	0 (0%)	2 (13%)	2 (12%)	4 (3.9%)	​
Tubulitis (t score)	​	​	​	​	​	<0.001
0	32 (60%)	0 (0%)	1 (6.7%)	8 (47%)	41 (40%)	​
1	14 (26%)	13 (72%)	2 (13%)	6 (35%)	35 (34%)	​
2	6 (11%)	5 (28%)	6 (40%)	1 (5.9%)	18 (17%)	​
3	1 (1.9%)	0 (0%)	6 (40%)	2 (12%)	9 (8.7%)	​
Intimal arteritis (v score)	​	​	​	​	​	<0.001
0	52 (100%)	18 (100%)	9 (60%)	14 (82%)	93 (91%)	​
1	0 (0%)	0 (0%)	2 (13%)	2 (12%)	4 (3.9%)	​
2	0 (0%)	0 (0%)	4 (27%)	1 (5.9%)	5 (4.9%)	​
Peritubular capillaritis (ptc score)	​	​	​	​	​	<0.001
0	51 (96%)	17 (94%)	9 (60%)	3 (18%)	80 (78%)	​
1	2 (3.8%)	1 (5.6%)	3 (20%)	5 (29%)	11 (11%)	​
2	0 (0%)	0 (0%)	2 (13%)	4 (24%)	6 (5.8%)	​
3	0 (0%)	0 (0%)	1 (6.7%)	5 (29%)	6 (5.8%)	​
c4d deposition in PTC	​	​	​	​	​	<0.001
0	53 (100%)	18 (100%)	14 (93%)	6 (38%)	91 (89%)	​
2	0 (0%)	0 (0%)	0 (0%)	2 (13%)	2 (2.0%)	​
3	0 (0%)	0 (0%)	1 (6.7%)	8 (50%)	9 (8.8%)	​
GBM double contours (cg score)	​	​	​	​	​	<0.001
0	52 (98%)	17 (94%)	15 (100%)	9 (53%)	93 (90%)	​
1	1 (1.9%)	1 (5.6%)	0 (0%)	3 (18%)	5 (4.9%)	​
2	0 (0%)	0 (0%)	0 (0%)	2 (12%)	2 (1.9%)	​
3	0 (0%)	0 (0%)	0 (0%)	3 (18%)	3 (2.9%)	​
Interstitial fibrosis (ci score)	​	​	​	​	​	<0.001
0	35 (66%)	6 (33%)	3 (20%)	4 (24%)	48 (47%)	​
1	17 (32%)	9 (50%)	12 (80%)	12 (71%)	50 (49%)	​
2	0 (0%)	3 (17%)	0 (0%)	1 (5.9%)	4 (3.9%)	​
3	1 (1.9%)	0 (0%)	0 (0%)	0 (0%)	1 (1.0%)	​
Tubular atrophy (ct score)	​	​	​	​	​	0.002
0	25 (47%)	1 (5.6%)	3 (20%)	3 (18%)	32 (31%)	​
1	27 (51%)	16 (89%)	12 (80%)	12 (71%)	67 (65%)	​
2	0 (0%)	1 (5.6%)	0 (0%)	2 (12%)	3 (2.9%)	​
3	1 (1.9%)	0 (0%)	0 (0%)	0 (0%)	1 (1.0%)	​
Arterial intimal thickening (cv score)	​	​	​	​	​	0.4
0	20 (40%)	4 (24%)	3 (20%)	5 (31%)	32 (33%)	​
1	19 (38%)	5 (29%)	8 (53%)	8 (50%)	40 (41%)	​
2	10 (20%)	7 (41%)	3 (20%)	2 (13%)	22 (22%)	​
3	1 (2.0%)	1 (5.9%)	1 (6.7%)	1 (6.3%)	4 (4.1%)	​
Arteriolar hyalinosis (ah score)	​	​	​	​	​	0.069
0	29 (55%)	8 (44%)	6 (40%)	4 (24%)	47 (46%)	​
1	20 (38%)	8 (44%)	6 (40%)	6 (35%)	40 (39%)	​
2	4 (7.5%)	1 (5.6%)	2 (13%)	5 (29%)	12 (12%)	​
3	0 (0%)	1 (5.6%)	1 (6.7%)	2 (12%)	4 (3.9%)	​

^a^ Median [Q1, Q3]; n (%).

^b^ Kruskal-Wallis rank sum test; Pearson’s Chi-squared test; Fisher’s exact test; NA.

^c^ dd-cf DNA >0.5%.

Absolute biomarker levels for urine CXCL10 (pg/mL), serum creatinine (μmol/L), and plasma dd-cfDNA (%) are compared among the four major diagnostic classifications (no rejection, borderline TCMR, Banff ≥1A TCMR, and AMR). The percentage of patients positive for urine CXCL10 and %dd-cfDNA are based on pre-defined thresholds. The subclinical rejection threshold used for urine CXCL10 was >13 pg/mL for male recipients over 2 weeks post-transplant, >33 pg/mL for female recipients from 2 weeks to 5 months post-transplant, and >13 pg/mL for female recipients >6 months post-transplant. An injury threshold of 0.5% dd-cfDNA was used. DSA is presented as a categorical variable with positivity defined as a minimum of 1000 MFI units of the immunodominant DSA.

### Association With Acute and Chronic Banff Scores

**FIGURE 3:**
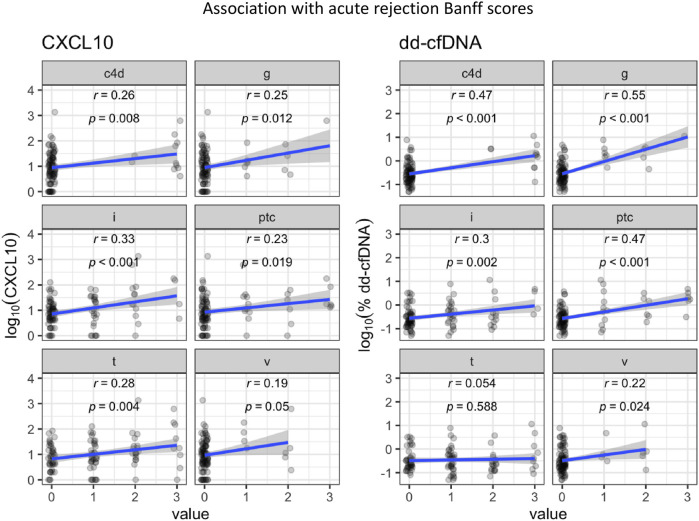
A comparison of the association of log-transformed biomarker levels (urine CXCL10 and plasma %dd-cfDNA) with the Banff scores required to diagnose acute rejection (g = glomerulitis, i = interstitial inflammation, ptc = peritubular capillaritis, t = tubulitis, and v = intimal arteritis).

Next, we sought to investigate the correlation between Banff scores and urine CXCL10 and plasma dd-cfDNA, respectively (Figure 3). Overall, log_10_%dd-cfDNA correlated more strongly than log_10_CXCL10 with glomerulitis (r = 0.55, p < 0.001 vs. r = 0.25, p = 0.012), peritubular capillaritis (r = 0.47, p < 0.001 vs. r = 0.23, p = 0.019), peritubular capillary c4d staining (r = 0.47, p < 0.001 vs. r = 0.26, p = 0.008), and glomerular basement membrane double contours (r = 0.41, p < 0.001 vs. r = 0.12, p = 0.226). In contrast, although neither log_10_%dd-cfDNA nor log_10_CXCL10 correlated strongly with tubulitis, performance characteristics favored log_10_CXCL10 (r = 0.28, p = 0.004 vs. r = 0.054, p = 0.588). The correlation was similar when interstitial inflammation scores were compared (Figure 3). In contrast, there was poor correlation with both log_10_%dd-cfDNA and log_10_CXCL10 and chronic Banff scores (Supplementary Figure S2).

### AMR vs. No Rejection

**FIGURE 4:**
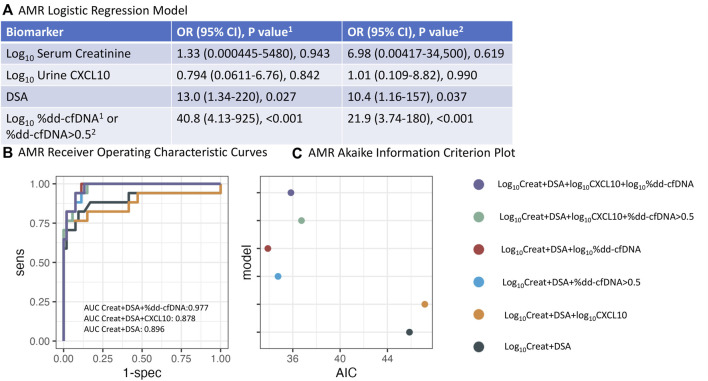
Logistic regression analysis for AMR. **(A)** Odds ratio for log_10_serum creatinine, log_10_urine CXCL10, DSA, and either log_10_%dd-cfDNA or %dd-cfDNA >0.5 using Firths penalized likelihood method. **(B)** Concomitant Receiver Operating Characteristic (ROC) analysis using various combinations of biomarkers. **(C)** Concomitant Akaike Information Criterion (AIC) analysis. (Creat = creatinine).

Because of the small number of AMR biopsies, the multi-predictor model for AMR vs. NR was fit using Firth’s penalized likelihood method (Figure 4). The log_10_ serum creatinine OR was 1.33 (95% CI 0.000445 to 5480, p = 0.943), log_10_CXCL10 OR 0.794 (95% CI 0.0611 to 6.76, p = 0.842), DSA OR 13.0 (95% CI 1.34 to 220, p = 0.027), and log_10_%dd-cfDNA OR 40.8 (95% CI 4.13 to 925, p < 0.001). When %dd-cfDNA was used as a categorical variable, the log_10_ serum creatinine OR was 6.98 (95% CI 0.00417 to 34,500, p = 0.619), log_10_CXCL10 OR 1.01 (95% CI 0.109 to 8.82, p = 0.990), DSA OR 10.4 (95% CI 1.16 to 157, p = 0.037), and dd-cfDNA >0.5% OR 21.9 (95% CI 3.74 to 180, p < 0.001). These data demonstrated that measurement of %dd-cfDNA improved AMR diagnosis independent of DSA.

Furthermore, AUCs for distinguishing AMR vs. NR increased from 0.696 (95% CI 0.527–0.865) for log_10_ serum creatinine alone to 0.967 (95% CI 0.930–1.000) and 0.959 (0.915–1.000) when log_10_ serum creatinine and either log_10_%dd-cfDNA or dd-cfDNA >0.5% were combined, respectively (Table 3). In contrast, the AUC for detecting AMR was 0.706 (95% CI 0.544–0.867) when log_10_ serum creatinine and log_10_CXCL10 were combined.

**TABLE 3:** Comparison of AUCs with 95% confidence intervals for all models of AMR, Banff ≥1A TCMR, and BPAR (Banff ≥1A TCMR and AMR), with percent dd-cfDNA included as either a continuous variable or applying a threshold of 0.5%.

Model	AMR (95%CI)	TCMR (95%CI)	BPAR (95%CI)
Log_10_serum creatinine	0.696 (0.527–0.865)	0.621 (0.452–0.789)	0.661 (0.534–0.787)
DSA	0.844 (0.730–0.957)	​	0.678 (0.591–0.765)
%dd-cfDNA >0.5	0.913 (0.828–0.998)	0.638 (0.510–0.766)	0.784 (0.693–0.875)
Log_10_ (%dd-cfDNA)	0.973 (0.941–1.000)	0.672 (0.493–0.851)	0.832 (0.729–0.934)
Log_10_ (cxcl10)	0.691 (0.540–0.841)	0.654 (0.482–0.826)	0.674 (0.552–0.796)
Log_10_ serum creatinine + DSA	0.896 (0.774–1.000)	​	0.763 (0.649–0.878)
Log_10_serum creatinine + %dd-cfDNA >0.5	0.959 (0.915–1.000)	0.724 (0.565–0.883)	0.849 (0.756–0.941)
Log_10_serum creatinine + log_10_ (%dd-cfDNA)	0.967 (0.930–1.000)	0.682 (0.497–0.867)	0.833 (0.729–0.937)
Log_10_serum creatinine + log_10_ (cxcl10)	0.706 (0.544–0.867)	0.717 (0.550–0.884)	0.713 (0.592–0.834)
DSA + %dd-cfDNA >0.5	0.932 (0.847–1.000)	​	0.791 (0.699–0.883)
DSA + log_10_ (%dd-cfDNA)	0.979 (0.954–1.000)	​	0.830 (0.728–0.932)
DSA + log_10_ (cxcl10)	0.851 (0.705–0.996)	​	0.750 (0.631–0.869)
Log_10_ (cxcl10) + %dd-cfDNA >0.5	0.920 (0.808–1.000)	0.725 (0.560–0.889)	0.828 (0.724–0.933)
Log_10_ (cxcl10) + log_10_ (%dd-cfDNA)	0.972 (0.940–1.000)	0.688 (0.516–0.860)	0.831 (0.729–0.934)
Log_10_ (cxcl10) + DSA + %dd-cfDNA >0.5	0.931 (0.820–1.000)	​	0.829 (0.724–0.934)
Log_10_ (cxcl10) + DSA + log_10_ (%dd-cfDNA)	0.979 (0.953–1.000)	​	0.829 (0.725–0.933)
Log_10_serum creatinine + DSA + %dd-cfDNA >0.5	0.977 (0.949–1.000)	​	0.855 (0.765–0.945)
Log_10_serum creatinine + DSA + log_10_ (%dd-cfDNA)	0.981 (0.957–1.000)	​	0.832 (0.729–0.935
Log_10_serum creatinine + log_10_ (cxcl10) + DSA	0.878 (0.748–1.000)	​	0.793 (0.682–0.904)
Log_10_serum creatinine + log_10_ (cxcl10) + %dd-cfDNA >0.5	0.959 (0.915–1.000)	0.767 (0.604–0.931)	0.862 (0.771–0.953
Log_10_serum creatinine + log_10_ (cxcl10) + log_10_ (%dd-cfDNA)	0.967 (0.930–1.000)	0.725 (0.554–0.896)	0.831 (0.727–0.936)
Log_10_serum creatinine + log_10_ (cxcl10) + DSA + %dd-cfDNA >0.5	0.978 (0.951–1.000)	​	0.866 (0.775–0.956
Log_10_serum creatinine + log_10_ (cxcl10) + DSA + log_10_ (%dd-cfDNA)	0.980 (0.955–1.000)	​	0.826 (0.720–0.933

### Banff ≥1A TCMR Vs. No Rejection

**FIGURE 5:**
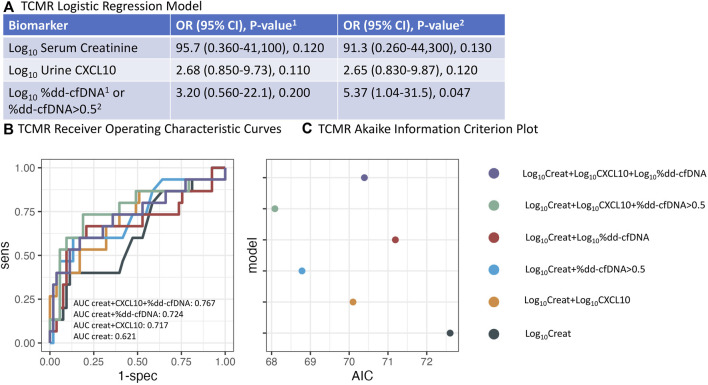
Logistic regression analysis for Banff ≥1A TCMR. **(A)** Odds ratio for log_10_serum creatinine, log_10_urine CXCL10, and either log_10_%dd-cfDNA or %dd-cfDNA >0.5 using logistic regression. **(B)** Concomitant Receiver Operating Characteristic (ROC) analysis using various combinations of biomarkers. **(C)** Concomitant Akaike Information Criterion (AIC) analysis. (Creat = creatinine).

Next, we compared the performance of serum creatinine, %dd-cfDNA, and urine CXCL10 in the detection of Banff ≥1A TCMR. AUCs and AICs were compared (Figure 5; Table 3). The log_10_ serum creatinine OR was 95.7 (95% CI 0.36 to 41,100, p = 0.120), log_10_CXCL10 OR 2.68 (95% CI 0.850 to 9.73, p = 0.110), and log_10_%dd-cfDNA OR 3.20 (95% CI 0.560 to 22.1, p = 0.200). When the 0.5% dd-cfDNA threshold was used, the log_10_ serum creatinine OR was 91.3 (95% CI 0.260 to 44,300, p = 0.130), log_10_CXCL10 OR 2.65 (95% CI 0.830 to 9.87, p = 0.120), and dd-cfDNA >0.5% OR 5.37 (95% CI 1.04 to 31.5, p = 0.047). Therefore, dd-cfDNA >0.5% was the only independent predictor of Banff ≥1A TCMR in this cohort. However, in a model without %dd-cfDNA, log_10_CXCL10 detected TCMR independently of serum creatinine (OR 3.12, 95% CI 1.09–10.4, p = 0.043) (data not shown).

AUCs for distinguishing TCMR vs. NR increased from 0.621 (95% CI 0.452–0.789) for log_10_ serum creatinine alone to 0.717 (95% CI 0.550–0.884) when log_10_ serum creatinine and log_10_CXCL10 were combined (Table 3). The AUC increased to 0.682 (95% CI 0.497–0.867) and 0.724 (95% CI 0.565–0.883) when log_10_ serum creatinine and either log_10_%dd-cfDNA or dd-cfDNA >0.5% were combined, respectively. When log_10_CXCL10 and either log_10_%dd-cfDNA or dd-cfDNA >0.5% were added to log_10_ serum creatinine, AUC increased to 0.725 (95% CI 0.554–0.896) and 0.767 (95% CI 0.604–0.931), respectively. That is, there was an improvement in the diagnosis of Banff ≥1A TCMR with the addition of both urine CXCL10 and %dd-cfDNA to serum creatinine.

### BPAR vs. No Rejection

**FIGURE 6:**
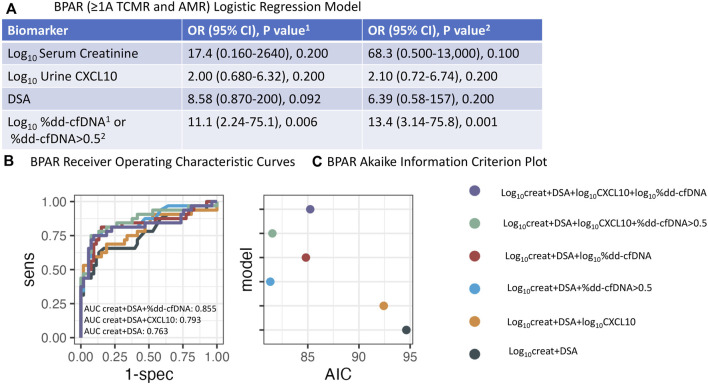
Logistic regression analysis for BPAR (Banff ≥1A TCMR and AMR). **(A)** Odds ratio for log_10_serum creatinine, log_10_urine CXCL10, DSA, and either log_10_%dd-cfDNA or %dd-cfDNA >0.5 using logistic regression. **(B)** Concomitant Receiver Operating Characteristic (ROC) analysis using various combinations of biomarkers. **(C)** Concomitant Akaike Information Criterion (AIC) analysis. (Creat = creatinine).

We also assessed performance in the diagnosis of BPAR (AMR and Banff ≥1A TCMR) vs. NR. Models were fit using various combinations (Figure 6; Table 3). In a multi-predictor logistic regression model, the log_10_ serum creatinine OR was 17.4 (95% CI 0.160–2640, p = 0.200), log_10_CXCL10 OR 2.00 (95% CI 0.680–6.32, p = 0.200), DSA OR 8.58 (95% CI 0.870–200, p = 0.092), and log_10_%dd-cfDNA OR 11.1 (95% CI 2.24–75.1, p = 0.006). Similarly, when dichotomized, dd-cfDNA >0.5% OR 13.4 (95% CI 3.14–75.8, p = 0.001) remained the only significant predictor of BPAR.

AUCs for distinguishing BPAR vs. NR increased from 0.661 (95% CI 0.534–0.787) for log_10_ serum creatinine alone to 0.833 (95% CI 0.729–0.937) and 0.849 (95% CI 0.756–0.941) when log_10_ serum creatinine and either log_10_%dd-cfDNA or dd-cfDNA >0.5% were combined, respectively (Table 3). AUC increased to 0.713 (95% CI 0.592–0.834) when log_10_CXCL10 was added to log_10_ serum creatinine.

### Borderline TCMR vs. No Rejection

**FIGURE 7:**
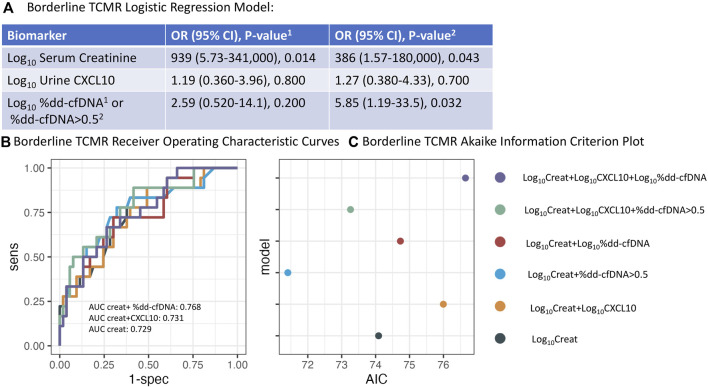
Logistic regression analysis for Borderline TCMR. **(A)** Odds ratio for log_10_serum creatinine, log_10_urine CXCL10, and either log_10_%dd-cfDNA or %dd-cfDNA >0.5 using logistic regression. **(B)** Concomitant Receiver Operating Characteristic (ROC) analysis using various combinations of biomarkers. **(C)** Concomitant Akaike Information Criterion (AIC) analysis. (Creat = creatinine).

Similar analyses were performed comparing borderline TCMR vs. NR. In a multi-predictor logistic regression model, the log_10_ serum creatinine OR was 939 (95% CI 5.43–309732, p = 0.014), log_10_CXCL10 OR 1.19 (95% CI 0.360–3.96, p = 0.800), and log_10_%dd-cfDNA OR 2.59 (95% CI 0.520–14.1, p = 0.200) (Figure 7). When the 0.5% dd-cfDNA threshold was used, the log_10_ serum creatinine OR was 386 (95% CI 1.57–180,000, p = 0.043), log_10_CXCL10 OR 1.27 (95% CI 0.380–4.33, p = 0.700), and dd-cfDNA >0.5% OR 5.85 (95% CI 1.19–33.5, p = 0.032). In this model, dd-cfDNA >0.5% was a significant independent predictor of borderline TCMR.

AUCs for distinguishing borderline TCMR vs. NR increased from 0.729 (95% CI 0.590–0.868) for log_10_ serum creatinine alone to 0.768 (95% CI 0.631–0.906) when log_10_ serum creatinine and dd-cfDNA >0.5% were combined (Table 4).

**TABLE 4:** Comparison of AUCs with 95% confidence intervals for all models of borderline TCMR with percent dd-cfDNA included as either a continuous variable or applying a threshold of 0.5%.

Model	AUC	95% CI
Log_10_serum creatinine	0.729	0.590–0.868
%dd-cfDNA >0.5	0.666	0.546–0.786
Log_10_ (% dd-cfDNA)	0.661	0.486–0.836
Log_10_(CXCL10)	0.508	0.329–0.687
Log_10_serum creatinine + %dd- cfDNA >0.5	0.768	0.631–0.906
Log_10_serum creatinine + log_10_ (%dd-cfDNA)	0.737	0.603–0.871
Log_10_serum creatinine + log_10_(CXCL10)	0.731	0.595–0.866
Log_10_(CXCL10) + % dd-cfDNA >0.5	0.724	0.573–0.876
Log_10_(CXCL10) + log_10_ (% dd-cfDNA)	0.647	0.471–0.822
Log_10_serum creatinine + log_10_(CXCL10) + % dd-cfDNA >0.5	0.781	0.654–0.908
Log_10_serum creatinine + log_10_(CXCL10) + log_10_ (%dd- cfDNA)	0.753	0.627–0.879

### Sensitivity, Specificity and Bivariate Scatter Plot for CXCL10 and dd-cfDNA

**FIGURE 8:**
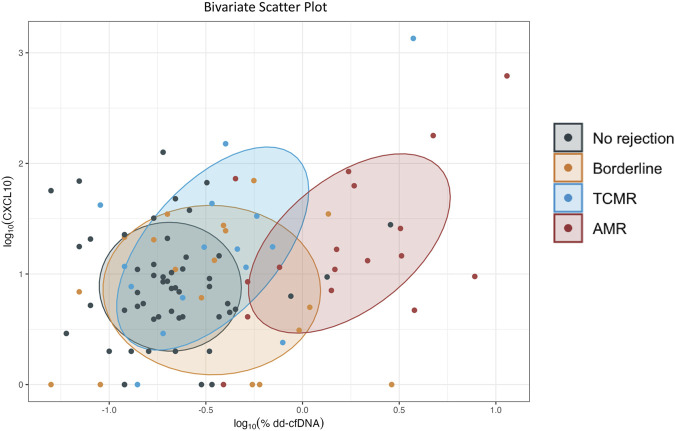
Bivariate scatter plot of log_10_ urine CXCL10 versus log_10_ %dd-cfDNA for no rejection, borderline TCMR, Banff ≥1A TCMR, and AMR. Ellipses are centered at the mean values in each target group.

Sensitivities and specificities, using the aforementioned thresholds, were calculated for each biomarker alone as well as together, if either were positive, for AMR, Banff ≥1A TCMR, and borderline TCMR (see Table 5). While performance characteristics for AMR favored %dd-cfDNA alone, sensitivity for both TCMR and borderline TCMR increased when urine CXCL10 was added to %dd-cfDNA. Sensitivity increased from 0.33 to 0.67 for TCMR and from 0.39 to 0.72 for borderline TCMR. Given each diagnostic category was compared to NR, specificities did not change across groups. A bivariate scatter plot of log_10_CXCL10 vs. log_10_%dd-cfDNA, with ellipses centered at the mean values for each group, for AMR, Banff ≥1A TCMR, borderline TCMR, and no rejection (see Figure 8) corroborates these observations, with differences associated with AMR favoring dd-cfDNA. For differences associated with Banff ≥1A TCMR, both urine CXCL10 and dd-cfDNA performed modestly. Differences associated with borderline TCMR slightly favored dd-cfDNA.

**TABLE 5:** Sensitivity and specificity for %dd-cfDNA and urine CXCL10 in AMR, Banff ≥1A TCMR, and borderline TCMR.

AMR vs. NR	Sensitivity	Specificity
%dd-cfDNA positive	0.88 (0.64–0.99)	0.94 (0.84–0.99)
CXCL10 positive	0.53 (0.28–0.77)	0.75 (0.62–0.86)
%dd-cfDNA or CXCL10 positive	0.94 (0.71–1.00)	0.72 (0.58–0.83)

TCMR vs. NR	Sensitivity
%dd-cfDNA positive	0.33 (0.12–0.62)
CXCL10 positive	0.53 (0.27–0.79)
%dd-cfDNA or CXCL10 positive	0.67 (0.38–0.88)

Borderline vs. NR	Sensitivity
%dd-cfDNA positive	0.39 (0.17–0.64)
CXCL10 positive	0.44 (0.22–0.69)
%dd-cfDNA or CXCL10 positive	0.72 (0.47–0.90)

The percentage of patients positive for urine CXCL10 and dd-cfDNA are based on pre-defined thresholds. The subclinical rejection threshold used for urine CXCL10 was >13 pg/mL for male recipients over 2 weeks post-transplant, >33 pg/mL for female recipients from 2 weeks to 5 months post-transplant, and >13 pg/mL for female recipients >6 months post-transplant. A threshold of 0.5% dd-cfDNA was used.

## Discussion

In this case-controlled study, we have shown that %dd-cfDNA is a better non-invasive biomarker of AMR than serum creatinine and urine CXCL10. However, when Banff ≥1A TCMR was assessed, %dd-cfDNA and urine CXCL10 were synergistic. Although performance characteristics for TCMR slightly favored %dd-cfDNA in our logistic regression model, their combination enhanced sensitivity and AUC. Over the last 15 years, various non-invasive diagnostic biomarkers of kidney transplant rejection have been evaluated, with the most data on urine chemokines, plasma dd-cfDNA, and blood gene expression profiling [13, 44]. Positive predictive values have remained low due to the low incidence of rejection under modern immunosuppression, the inherent limitations of histological sampling which may underestimate rejection, and confounders such as infection and non-alloimmune acute kidney injury. As a result, they appear to be more promising as non-invasive “rule out” tests for rejection in stable patients rather than as stand-alone diagnostic tests. At the same time, further study on how these tests may be used as adjuncts to histology are necessary.

Despite advances in our understanding of the diagnostic accuracy of urine CXCL10 and dd-cfDNA in kidney transplant rejection, few studies [45] have simultaneously measured these two biomarkers in the same cohort. In this study we showed urine CXCL10 was a better correlate of tubulitis than %dd-cfDNA and, when compared to serum creatinine alone, independently improved the non-invasive diagnosis of Banff ≥1A TCMR but did not add to AMR detection. Conversely, plasma %dd-cfDNA correlated more strongly than urine CXCL10 with microvascular inflammation and independently improved the non-invasive diagnosis of both AMR and Banff ≥1A TCMR. Overall, our histology correlations are consistent with what others have reported for both %dd-cfDNA and urine CXCL10 [20, 29, 46]. Although Rabant et al. demonstrated that urine CXCL10 independently improves AMR diagnosis, %dd-cfDNA was not included in that analysis [47]. Furthermore, the AUC for AMR in this study, when compared to %dd-cfDNA performance, was relatively modest at 0.702.

Overall, this study—the first to simultaneously measure these two novel biomarkers in a meticulously characterized clinical-pathological cohort including protocol biopsies—demonstrates that urine CXCL10 may not be helpful as a diagnostic biomarker of AMR when plasma %dd-cfDNA and DSA are available. Our study also adds to prior work demonstrating that %dd-cfDNA improves the non-invasive detection of AMR independently of serum creatinine and DSA [10, 39].

In contrast, both urine CXCL10 and %dd-cfDNA were modest predictors of Banff ≥1A TCMR. These data are consistent with recent studies showing modest performance for both urine CXCL10 and %dd-cfDNA for detecting borderline TCMR and/or Banff ≥1A TCMR [28, 35, 36, 48]. The large proportion of TCMR without vascular lesions in our study may explain the lower %dd-cfDNA diagnostic performance compared to other studies [29]. The decrease in performance of urine CXCL10 when %dd-cfDNA was included in our multivariable analysis suggests some diagnostic overlap. This may be explained by the fact that both correlate similarly with interstitial inflammation (Figure 3). In addition, the inclusion of biopsies with low-grade inflammation in the NR group, 40% of which had tubulitis scores ≥ t1 and would have met the 2015 definition of borderline, although appropriate, may have decreased the performance characteristics of urine CXCL10. Nonetheless, its greater sensitivity compared to %dd-cfDNA for both Banff ≥1A and borderline TCMR supports its potential as a screening tool. Although other diagnostic advantages of urine CXCL10 may lie in its higher correlation with tubulitis, its early identification of post-transplant infections such as BK and ease of sample collection, in our study, %dd-cfDNA, due to its higher specificity, more effectively categorized low-grade inconsequential inflammation such as isolated tubulitis as no rejection [15, 16]. As a result, one approach might be to use urine CXCL10 and %dd-cfDNA sequentially to more accurately non-invasively detect Banff ≥1A and borderline TCMR. Given that both of these rejection phenotypes are clinically important due to the risk of developing persistent chronic active TCMR, *de novo* DSA, or AMR [49–51], defining optimally targeted screening strategies remains a priority.

Limitations of our study include potential over-estimation of diagnostic performance based on a curated case-control cohort with exclusion of non-rejection diagnoses. The cohort consisted of 47% clinical indication biopsies wherein the decision to biopsy was already made using serum creatinine and clinical judgement. Furthermore, as centers that perform protocol biopsies, there is a relative over-representation of more severe rejection phenotypes (AMR and Banff ≥1A TCMR) compared to borderline TCMR. While borderline TCMR is the most common rejection phenotype we see within the first year (unpublished data), AMR is less frequent yet increases over time. In addition, while urine CXCL10 was selected due to having an assay with well-developed performance characteristics and pre-analytical sample handling data and the potential to be used in clinical labs [37], exclusion of urine CXCL9, a urine chemokine with more favorable diagnostic characteristics [48] is a limitation. Further, due to the small size of our cohort, we were unable to attempt to distinguish among AMR subsets, e.g., our AMR group includes a heterogeneous mix of mixed rejection as well as DSA negative, c4d negative microvascular inflammation (MVI). We do know that these phenotypes have different long-term prognoses and that performance characteristics of the biomarkers may differ [52]. Finally, this exploratory study was not designed or powered to assess whether absolute biomarker levels reflect degree of injury and whether changes post-therapy reflect resolution vs. persistent inflammation. Nevertheless, this analysis offers new and important insights into the relative associations of urine CXCL10 and plasma dd-cfDNA with different types of rejection.

Taken together, the potential for individualized biomarker-guided screening strategies for AMR and TCMR deserves further investigation in larger unselected prospective observational cohort studies to provide real-world diagnostic performance characteristics. Such studies will enhance practical clinical implementation of these biomarkers into post-transplant care [44, 53].

## Data Availability

The raw data supporting the conclusions of this article will be made available by the authors, without undue reservation.
