# Supplementary figures and images for: Diagnostic Potential of Urine CXCL10 and Donor-Derived cfDNA in Kidney Transplant Rejection
Source: Transpl Int. 2026 Mar 27;39:15517. doi: 10.3389/ti.2026.15517 (PMC13067146; doi:10.3389/ti.2026.15517)

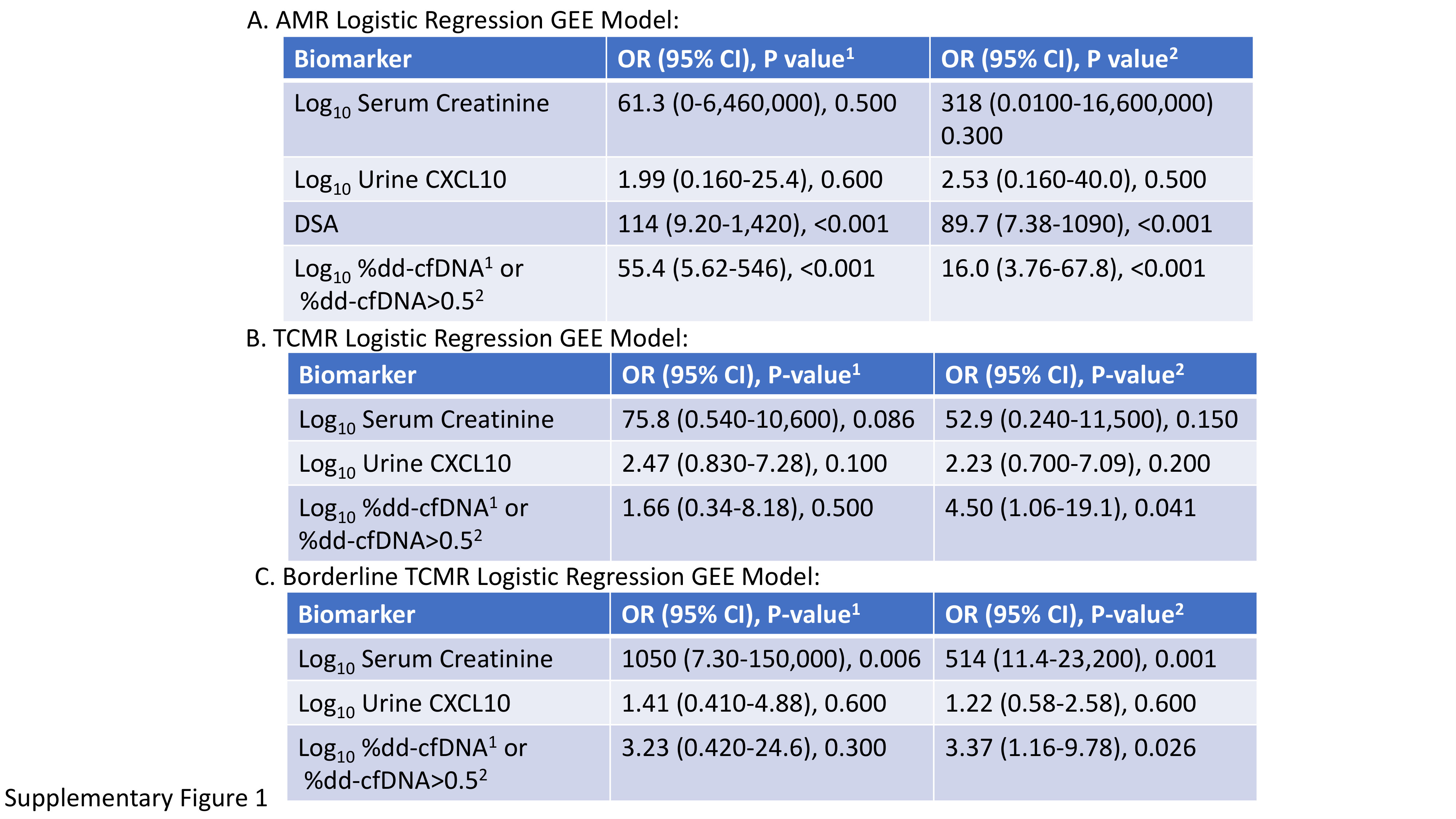

Supplement: Supplementary file 1 [file Image1.jpeg]

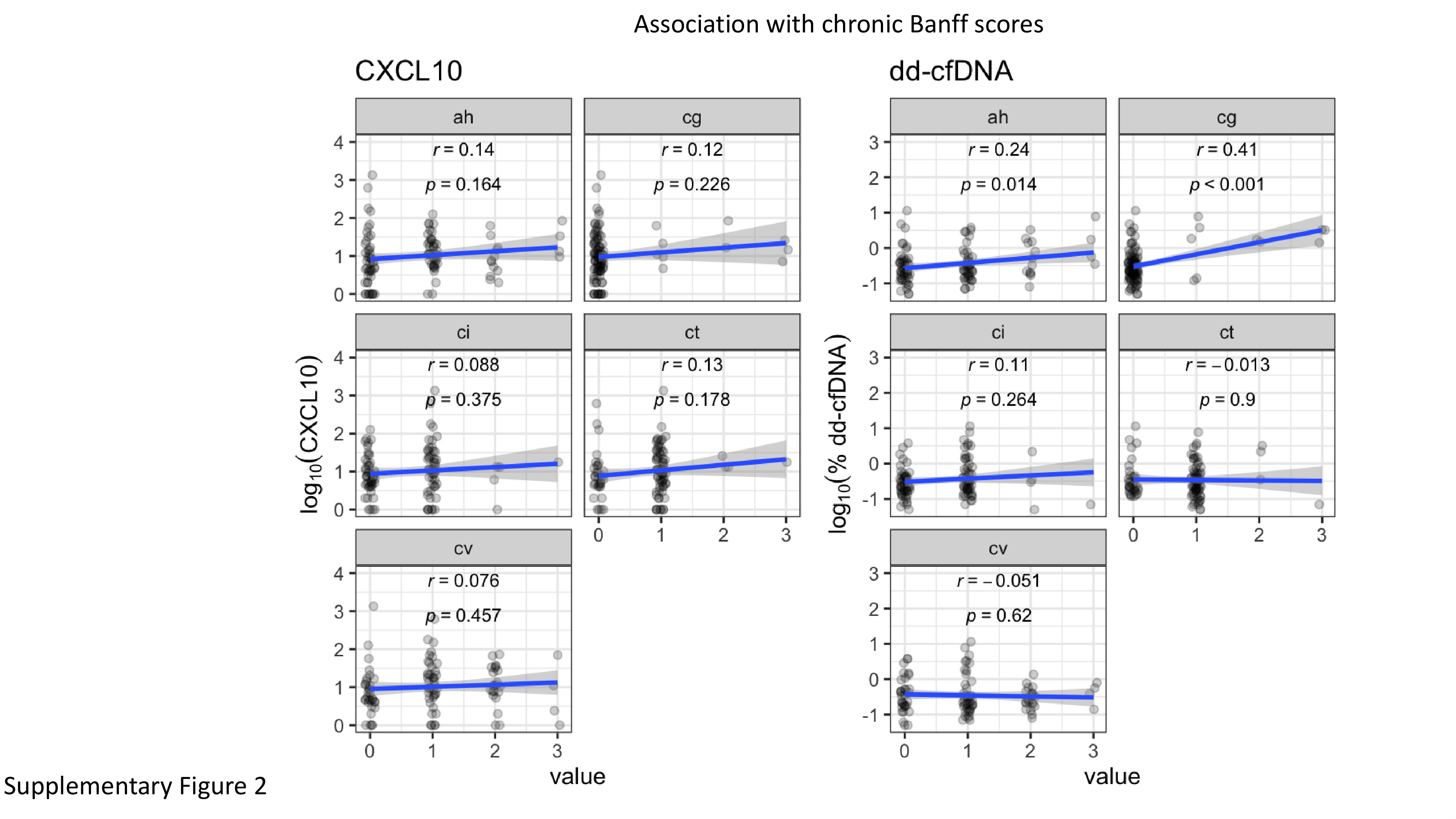

Supplement: Supplementary file 2 [file Image2.jpeg]
