# Supplementary material for: Diagnostic Potential of Urine CXCL10 and Donor-Derived cfDNA in Kidney Transplant Rejection
Source: Transpl Int. 2026 Mar 27;39:15517. doi: 10.3389/ti.2026.15517 (PMC13067146; doi:10.3389/ti.2026.15517)
Supplement: Supplementary file 3 [file Table1.doc]

Supplementary table and figure legends and supplementary tables:

Supplementary Table 1. Demographic characteristics of the selected and excluded cohort.

Supplementary Table 2. Diagnostic categories comparing all biopsies versus only first

biopsies versus only last biopsies.

Supplementary Table 3. HLA mismatch at the A, B and DR loci of the overall selected cohort and by diagnostic subgroup (no rejection, borderline TCMR, Banff≥1A TCMR and AMR).

Supplementary Figure 1: Sensitivity analyses using a generalized estimating equation model to account for multiple biopsies per patient. Odds ratios for log_10_serum creatinine, log_10_urine CXCL10 and either log_10_%dd-cfDNA or %dd-cfDNA>0.5 using logistic regression for A. AMR B. Banff≥1A TCMR and C. Borderline TCMR. DSA is only included in the AMR logistic regression model.

Supplementary Figure 2: A comparison of the association of log-transformed biomarker levels (urine CXCL10 and %dd-cfDNA) with chronic Banff scores (ah=arteriolar hyalinosis, cg=glomerular basement membrane double contours, ci=interstitial fibrosis, ct=tubular atrophy, cv=vascular fibrous intimal thickening)

Supplementary Table 1. Demographic characteristics of the included and excluded cohort.

| **Characteristic** | **Included** N = 103*^1^* | **Excluded** N = 921*^1^* | **p-value***^2^* |
| --- | --- | --- | --- |
| Recipient age at biopsy | 52 [40, 61] | 53 [43, 62] | 0.4 |
| Male sex | 72 (70%) | 580 (63%) | 0.2 |
| Cause of ESRD |  |  | 0.006 |
| Polycystic kidney disease | 26 (29%) | 111 (13%) |  |
| Glomerulonephritis | 25 (28%) | 313 (38%) |  |
| Diabetes | 18 (20%) | 142 (17%) |  |
| Vascular | 8 (9.0%) | 100 (12%) |  |
| Genetic | 5 (5.6%) | 38 (4.6%) |  |
| Other | 7 (7.9%) | 123 (15%) |  |
| Unknown | 14 (13.6%) | 94 (10.2%) |  |
| Dialysis duration (months) | 31 [13, 59] | 39 [17, 72] | 0.024 |
| Repeat transplant | 5 (4.9%) | 98 (10.6%) | 0.064 |
| Donor age | 51 [36, 57] | 53 [40, 60] | 0.13 |
| Donor male sex | 53 (51%) | 502 (55%) | 0.5 |
| Living donor | 25 (24%) | 182 (20%) | 0.3 |
| Delayed graft function | 13 (13%) | 156 (17%) | 0.3 |
| Induction therapy |  |  | <0.001 |
| Basiliximab | 79 (77%) | 719 (78%) |  |
| Anti-thymocyte globulin (ATG) | 13 (13%) | 185 (20%) |  |
| Other | 10 (9.8%) | 14 (1.5%) |  |
| *^1^* Median [Q1, Q3]; n (%) | | | |
| *^2^* Wilcoxon rank sum test; Pearson’s Chi-squared test; Fisher’s Exact Test for Count Data with simulated p-value (based on 2000 replicates); Fisher’s exact test | | | |

Supplementary Table 2. Diagnostic categories comparing all biopsies versus only first biopsies versus only last biopsies.

| **Category** | All Biopsies | First Biopsies | Last Biopsies |
| --- | --- | --- | --- |
|  | **N = 121***^1^* | **N = 103***^1^* | **N = 103***^1^* |
| No Rejection | 58 (48%) | 52 (50.5%) | 53 (51.5%) |
| Borderline | 19 (16%) | 16 (15.5%) | 18 (17.5%) |
| TCMR | 18 (15%) | 18 (17.5%) | 15 (14.6%) |
| AMR | 26 (21.5%) | 17 (16.5%) | 17 (16.5%) |

*^1^* n (%)

Supplementary Table 3. HLA mismatch at the A, B and DR loci of the overall selected cohort and by diagnostic subgroup (no rejection, borderline TCMR, Banff≥1A TCMR and AMR).

| **Characteristic** | **Overall**  N=103^1^ | **No Rejection** N = 53^1^ | **Borderline** N = 18^1^ | **TCMR**  N = 15^1^ | **AMR** N = 17^1^ | **p-value^2^** |
| --- | --- | --- | --- | --- | --- | --- |
| HLA A mismatch |  |  |  |  |  | 0.9 |
| 0 | 9 (8.7%) | 5 (9.4%) | 1 (5.6%) | 2 (13%) | 1 (5.9%) |  |
| 1 | 46 (45%) | 23 (43%) | 9 (50%) | 8 (53%) | 6 (35%) |  |
| 2 | 48 (47%) | 25 (47%) | 8 (44%) | 5 (33%) | 10 (59%) |  |
| HLA B mismatch |  |  |  |  |  | 0.7 |
| 0 | 8 (7.8%) | 6 (11%) | 1 (5.6%) | 0 (0%) | 1 (5.9%) |  |
| 1 | 35 (34%) | 16 (30%) | 6 (33%) | 8 (53%) | 5 (29%) |  |
| 2 | 60 (58%) | 31 (58%) | 11 (61%) | 7 (47%) | 11 (65%) |  |
| HLA DR mismatch |  |  |  |  |  | 0.072 |
| 0 | 17 (17%) | 9 (17%) | 3 (17%) | 4 (27%) | 1 (5.9%) |  |
| 1 | 53 (52%) | 32 (62%) | 6 (33%) | 8 (53%) | 7 (41%) |  |
| 2 | 32 (31%) | 11 (21%) | 9 (50%) | 3 (20%) | 9 (53%) |  |

| *^1^* n (%) |
| --- |
| *^2^* Kruskal-Wallis rank sum test; Fisher’s Exact Test for Count Data with simulated p-value (based on 2000 replicates); Pearson’s Chi-squared test |
